# Clinical outcome of SARS-CoV-2 infections occurring in multiple myeloma patients after vaccination and prophylaxis with tixagevimab/cilgavimab

**DOI:** 10.3389/fonc.2023.1157610

**Published:** 2023-03-29

**Authors:** Andrea Duminuco, Alessandra Romano, Dario Leotta, Enrico La Spina, Daniela Cambria, Anna Bulla, Vittorio Del Fabro, Daniele Tibullo, Cesarina Giallongo, Giuseppe A. Palumbo, Concetta Conticello, Francesco Di Raimondo

**Affiliations:** ^1^ Unità Operativa Complessa di Ematologia con Trapianto di Midollo Osseo, Azienda Ospedaliero-Universitaria Policlinico “G.Rodolico-San Marco”, Catania, Italy; ^2^ Dipartimento di Specialità Medico-Chirurgiche, CHIRMED, Sezione di Ematologia, Università degli Studi di Catania, Catania, Italy; ^3^ Dipartimento di Scienze Biomediche e Biotecnologie Avanzate, University of Catania, Catania, Italy; ^4^ Dipartimento di Scienze Mediche Chirurgiche e Tecnologie Avanzate “G.F. Ingrassia”, University of Catania, Catania, Italy

**Keywords:** COVID-19, vaccination, SARS-COV-2 spike antibody, multiple myeloma, tixagevimab/cilgavimab prophylaxis

## Abstract

**Introduction:**

Patients with multiple myeloma (MM) frequently reported immune impairment with an increased risk for infection-related mortality. We aimed to evaluate the immune response in MM patients vaccinated for SARS-CoV-2 during active treatment.

**Methods:**

We enrolled 158 patients affected by active MM or smoldering MM (SMM) and 40 healthy subjects. All subjects received 2 or 3 doses of the BNT162b2 (Pfizer/BioNTech) vaccine, and the anti-spike IgG values were evaluated after every dose. We applied the Propensity Score Matching (PSM) as a consequence of the limited sample size and its heterogeneity to adjust for differences in baseline clinical variables between MM patients who achieved or not a vaccine response after 2 or 3 doses.

**Results:**

At 30 days from the second dose, the median antibodies level in MM was 25.2 AU/mL, lower than in SMM and in the control group. The same results were confirmed after the third dose, with lower median anti-spike IgG levels in MM, compared to SMM and control group. Following PSM, lack of response to SARS-CoV-2 complete vaccination plus boost was associated with age more than 70 years old and use of high-dose of steroids. We failed to identify an association between specific treatment types and reduced vaccine response. The use of prophylaxis with tixagevimab/cilgavimab for 40 non-responder patients after 3 doses of vaccine has proven to be an effective and safe approach in reducing the risk of serious illness in the event of a breakthrough SARS-CoV-2 infection, faced with a mild symptomatic course, and in providing protection instead of long-term humoral immune vaccine responses. Following PSM, only the high-risk cytogenetic abnormalities were associated with an increased risk of developing a breakthrough SARS-CoV-2 infection.

**Conclusion:**

Monitoring the immune response is fundamental in MM patients that remain highly vulnerable to SARS-CoV-2 despite the vaccine. The use of prophylaxis with tixagevimab/cilgavimab can guarantee better protection from the severe form of the disease.

## 1 Introduction

Multiple myeloma (MM) is a malignancy caused by the uncontrolled proliferation of plasma cells. It represents the second most frequent hematological malignancy, accounting for 1% of all cancers (1), and over 2% of cancer deaths (2). Even though the precise cause of MM is still unknown, the knowledge so far acquired suggests that MM is a consequence of an abnormality during the process of B-cell maturation, such as somatic hypermutation or class switching. Genetic and environmental causes have a significant role in the development of MM (3).

Ideally, initial therapy for MM should allow rapid disease control and reversal of disease-related complications, be well tolerated with minimal and manageable toxicity, decrease the risk of early death, and guarantee a thriving collection of stem cells when a bone marrow transplant is considered as a therapeutic option (4).

Despite the recent introduction of novel anti-MM drugs, such as proteasome inhibitors (PIs), immunomodulatory (IMiDs) agents, and monoclonal antibodies (MoAbs), and the continuous change in treatment landscapes, MM remains an incurable disease. In addition, these several lines of therapies and the impairment of the immune system caused by the disease could increase the risk of infections (due to an impairment of the immune system). This complication represents a significant cause of morbidity and mortality in MM patients (5). A study conducted at the Peter MacCallum Cancer Centre (PMCC) and St Vincent’s Hospital in Patients with MM between January 2013 and December 2018 tried to determine patterns, risks, and outcomes of infections in patients with MM managed with second-generation therapies and mAbs. Among 148 patients with MM, 345 infection episodes were identified. The overall incidence rate of infection was 1.7 per patient-year (6). Almost all novel anti-MM drugs combined with steroids are characterized by effects on the immune system different from those of conventional anti-MM agents. These assumptions underline that subjects affected by MM are patients with a greater risk of developing infectious complications, which can prove fatal.

From the end of January 2020, infection by SARS-CoV-2 has radically changed our lives. Since the severe acute respiratory syndrome COVID-19 (SARS-CoV-2) pandemic is still ongoing, counting more than 6.24 million deaths (7), it is still to clarify why the response to infection differs from person to person and which immunopathological mechanisms lead to severe disease. Concerning this, it is well documented that patients with impairment of the immune system are more at risk of developing severe SARS-CoV-2 with reduced survival.

In this study, we investigated the immune response in MM patients vaccinated for SARS-CoV-2 during their active anti-MM treatment, evaluating the protection level from infection reached in this field. We try to identify which clinical or pharmacological factors can predict failure to respond to complete vaccination against SARS-CoV-2 plus booster dose in the era of immunotherapies that are now a milestone of neoplastic treatment.

## 2 Patients and methods

### 2.1 Study design and patients’ characteristics

The study included 133 adult multiple myeloma patients referred to our Division of Hematology at Policlinico “G. Rodolico-San Marco” in Catania, Italy, between March 15th, 2021, and August 15th, 2021. Participants meeting eligibility criteria were adults aged 18 or older undergoing treatment for MM at our outpatients’ service, candidates to receive 3 doses of BNT162b2 (Pfizer/BioNTech) vaccine and anti-MM treatment. The serum samples were collected before the first vaccination, 30 days after the second, and 60 days after the third dose. Per internal policy, all patients discontinued steroids seven days before and seven days after vaccine inoculation. We defined high-dose steroid-treated patients who took more than 40 mg weekly.

Control groups were: 1) 40 healthy subjects aged 18 or older who denied infection with or known exposure to SARS-CoV-2; 2) 25 asymptomatic patients referred to our Center for smoldering MM (SMM) not requiring treatment. Control subjects were screened to confirm negative exposure status to the virus through the SARS-CoV-2 RBD solid-phase sandwich ELISA test. Clinical records, laboratory findings, and physical examination records of healthy people were obtained and curated with a customized data collection form. Three investigators independently reviewed the data collection forms to verify data accuracy.

The SARS-CoV-2 infection diagnosis was confirmed by nasopharyngeal swab collection in accordance with local prevention guidelines. The study was approved by the Institutional Review Board (IRB) (Comitato etico Catania 1, https://www.policlinicovittorioemanuele.it/comitato-etico-catania-1, #CO.TIP. 34/2020/PO 0016693 released on 15 Apr 2020) and performed in accordance with the principles of the Declaration of Helsinki and the International Conference on Harmonization Good Clinical Practice guidelines.

### 2.2 Procedures

Health records of patients followed in our hospital were evaluated to capture the following information: details and timing of the tumor treatment and laboratory parameters at the time of the first, second, and third dose of SARS-CoV-2 vaccination. Active antineoplastic treatment was defined as receiving anticancer therapy within 15 days of the first SARS-CoV-2 vaccination.

Serum was obtained from 5 ml peripheral clotted blood after centrifugation at 900 x g [U5] and stored in aliquots at −20°C. All samples and data were de-identified following collection, and researchers conducting assays were blinded to clinical data until the final comparative analysis.

Immunogenicity assessment was determined using a chemiluminescent microparticle immunoassay (SARS-CoV-2 IgG II Quant assay on an ALINITY analyzer; Abbott) to quantify IgG antibodies from the patient’s plasma. The assay detects antibodies against the receptor binding protein of the SARS-CoV-2 spike protein’s S1 subunit. A value ≥40 arbitrary units per milliliter (AU/ml) was considered evidence of vaccination response.

### 2.3 Statistical analysis

Qualitative results were summarized in counts and percentages. Descriptive statistics were generated to analyze the results, and a p-value under 0.05 was considered significant.

Normality was verified using the Shapiro-Wilks test and graphically using the Q–Q plot. Continuous variables are expressed as median (IQR) since a preliminary analysis showed that data distribution was not expected. The Mann-Whitney U test or Kruskal–Wallis test was used to compare continuous data, and the chi-squared test or Fisher’s exact test to compare categorical data.

We applied the Propensity Score Matching (PSM) as consequence of the limited sample size and its heterogeneity to adjust for differences in baseline clinical variables between MM patients who achieved or not a vaccine response after 2 or 3 doses. The covariates balanced between groups were: age (used as a dichotomic variable, less than 70 years, equal to or more than 70 years old), biologic sex (female, male), lymphopenia [absolute lymphocyte count (ALC), ≤1000 versus >1000 per μl], cancer status (active or <VGPR versus not active or CR+VGPR), lines of treatment (first versus second or more), type of therapy (containing or not monoclonal antibodies), steroid exposure (used as a dichotomic variable, yes or not) and FISH risk (standard versus high; high risk was defined due to the presence of t(4,14), t(14;16), t(14;20), gain/amp(1q21), del(1p), and del(17p). In PSM, we selected the calliper 0.25 of the standard deviation for drawing the control units (non responder) to match the treated units (responder) with the nearest-neighbour method and a 2:1 ratio (non responder: responder). Due to the limited number of events, we considered variable selection in regression analysis by the elastic net regularization with a mixing parameter (LASSO).

All calculations were performed using MedCalc Statistical Software version 13.0.6 (MedCalc Software bvba, Ostend, Belgium; http://www.medcalc.org; 2014) and XLSTAT version 2021.5 - Life Sciences, released in December 2021.

## 3 Results

### 3.1 Patients and controls characteristics

A total of 158 patients affected by multiple myeloma (active/in treatment, N=133; smoldering MM and MGUS, N=25) and 40 healthy subjects were evaluated. All subjects received 2 doses of the BNT162b2 (Pfizer/BioNTech) vaccine at a 21-day interval. Healthy and SMM subjects received the third boost dose with BNT162b2 (Pfizer/BioNTech) at 90-day intervals. In the group of active MM, 121 patients received the boost dose; 12 MM patients died of disease progression before receiving the boost dose.

A detailed description of the clinical characteristics of MM, SMM, and healthy subjects is summarized in **Table 1**. The control group consisted of 40 health operators (median age 46 years, range 26-66) without IgM hypogammaglobulinemia, and all of them were vaccinated with seasonal flu vaccination before the anti-SARS-CoV2 complete cycle of immunization. The median age of patients with SMM was 60 years (range 52-72). Only one-third of SMM patients received seasonal flu vaccination before the anti-SARS-CoV2 full vaccination cycle. The median age of patients with active MM was 66 years (range 41-83), and more than half of them (59%) received seasonal flu vaccination before the anti-SARS-CoV2 full vaccination cycle.

**Table 1:** Clinical characteristics of subjects included in the study who received three doses of BNT162b2 (Pfizer-BioNTech) COVID vaccine.

	Healthy	SMM	MM
**Patients, n (%)**	40 (100)	25 (100)	133 (100)
**Age, years median (range)**	46 (26-66)	60 (52-72)	66 (41-83)
**Gender**			
Female, n (%)	26 (65)	9 (36)	55 (41)
**Previous vaccination against seasonal flu**			
Yes (%)	40 (100)	9 (36)	78 (59)
**IgM, mg/dL**			
median (range)	83 (60-130)	72 (53-92)	35 (6-150)
**Documented immunoparesis, n (%)**	0 (0)	2 (8)	61 (78)
**ANC, 10^3^cells/mmc**	4.4	4.3	3.0
median (range)	(2.8-7.2)	(3.3-12.5)	(1.1-13.3)
**ALC, 10^3^ cells/mmc**	2.8	2.6	1.3
median (range)	(1.5-4.3)	(1.0-5.9)	(0.3-2.5)
**AMC, 10^3^ cells/mmc**	0.6	0.4	0.5
median (range)	(0.3-0.9)	(0.3-0.8)	(0.1-2.8)
**FISH-high risk, (%)**	/	/	35 (26.3)
**For newly diagnosed MM,** **time-to-first treatment, days (IQR)**	/	/	15 [12-27]

SMM, smoldering myeloma; MM, noultiple myeloma; ANC, absolute neutrophil count; ALC, absolute lymphocyte count; AMC, absolute monocyte count.

Compared to SMM and healthy subjects, MM patients had a lower amount of IgM, but comparable amounts of absolute neutrophils count (ANC), absolute lymphocyte count (ALC), absolute monocyte count (AMC) and a lower rate of flu vaccination. Indeed, most MM patients had documented immunoparesis (78%), defined as IgM hypogammaglobulinemia, due to IgM concentration <50 mg/dL. Median absolute counts of neutrophils (3.0, range 1.1-13.3 x10^3^/mmc) and lymphocytes (1.3, range 0.3-2.5 x10^3^/mmc) in peripheral blood were lower in MM patients than in SMM and healthy subjects, without achieving statistical significance. 78/133 (59%) MM patients received seasonal flu vaccination before the anti-SARS-CoV2 complete vaccination cycle.

All MM patients were in active treatment, including 59 newly-diagnosed MM (with a median time-to-first treatment of 15 days, IQR 12-27), 32 relapsed-refractory MM, treated with a variety of therapies at the time of vaccination, and 42 patients with no active disease were receiving continuous therapy with daratumumab or lenalidomide at time of SARS-CoV-2 vaccination, as shown in **Table 2**. Most patients received monoclonal antibodies (MoAbs, daratumumab or elotuzumab) alone (n=24, 18%) or in combination with immunomodulatory agents (lenalidomide or pomalidomide, n=47, 36%). Since January 2022, the quadruplet of daratumumab, lenalidomide, bortezomib, and dexamethasone was available for newly diagnosed patients eligible for autologous stem cell transplantation (n=9, 6%). Other treatments included immunomodulatory agents (IA, 20%), alone or combined with proteasome inhibitors (PI, 11%). High-risk MM, defined as presence of cytogenetic abnormalities del(17p), t(4;14), t(14;16), amp1q, del(1p) (8), was reported in 35 patients (25.6%). There were no significant differences among the rate of seasonal anti-flu vaccination, documented immunoparesis, median IgM, ANC, ALC and AMC among the different treatment groups (data not shown).

**Table 2:** Clinical characteristics and treatment overview of 133 MM patients who received two BNT162b2 (Pfizer-BioNTech) SARS-CoV-2 vaccines.

	IA	PI	IA+PI	MoAbs	IA+ MoAbs	IA+PI MoAbs
**Patients, n (%)**	26 (20)	12 (9)	15 (11)	24 (18)	47 (36)	9 (6)
**Age, yrs median (range)**	62 (47-83)	62 (48-76)	61 (41-75)	69 (53-80)	69 (59-80)	69 (59-80)
Gender, Female, n	13	6	7	7	25	1
**Previous vaccination against seasonal flu**	15	7	6	14	29	7
**Disease Activity**						
CR+VGPR	22	5	11	14	26	6
PR or less	4	7	4	10	21	3
**Previous lines of treatment**						
0	19	4	3	0	25	9
1	5	5	10	12	18	0
2 or more	2	3	2	12	4	0
ASCT in the last 12 months	11	3	9	11	10	0
**FISH-high risk, (%)**	4 (3)	4 (3)	7 (5.3)	7 (5.3)	12 (9)	1 (0.8)
**IgM, mg/dL**	36	31	23	26	49	49
median (range)	(11-131)	(20-51)	(9-69)	(6-150)	(35-134)	(35-134)
**Documented immunoparesis, n**	16	7	12	17	39	8
**ANC, 10^3^ cells/mmc**	3.1	5.3	3.9	3.8	3.6	2.8
median (range)	(1.1-6.8)	(2.2-12.7)	(1.4-13.3)	(1.1-10.1)	(1.1-7.5)	(1.1-7.5)
**ALC, 10^3^ cells/mmc**	1.3	1.1	1.1	1.2	1.3	1.5
median (range)	(0.3-2.5)	(0.6-2.6)	(0.4-1.7)	(0.3-2.1)	(0.1-3.4)	(0.1-3.4)
**AMC, 10^3^ cells/mmc**	0.5	0.6	0.5	0.6	0.4	0.4
median (range)	(0.1-2.8)	(0.2-1.2)	(0.1-1.0)	(0.1-1.0)	(0.2-2.1)	(0.2-2.1)

IA, immunomodulatory agents; PI, proteasome inhibitor; MoAbs, monoclonal antibodies; CR, complete response; VGPR, very good partial R; PR, partial response; ASCT, autologous stem cell transplant; ANC, absolute neutrophil count; ALC, absolute lymphocyte count; AMC, absolute monocyte count.

### 3.2 Serological response to BNT162b2 (Pfizer-BioNTech) SARS-CoV-2 vaccination in MM patients

Three newly-diagnosed MM patients, resulted in IgG against SARS-CoV-2 nucleocapsid proteins (anti-N), proving a prior exposure to the virus at the time of the first vaccination (2906.7, 1333.9 and 6901.5 AU/ml). These three subjects showed a high antibody response after vaccination.

At 30 days from the second dose of BNT162b2 (Pfizer-BioNTech) SARS-CoV-2 vaccine, the median anti-spike IgG levels in MM was 25.2 (interquartile range, IQR, 2.8-135.6 AU/mL), significantly lower than those in SMM (360.2, IQR, 245-424 AU/mL, p<0.0001, Mann–Whitney U test) and in the control group (704, IQR, 390-1340 AU/mL, p<0.0001, Mann–Whitney U test).

At 60 days from the third dose of BNT162b2 (Pfizer-BioNTech) SARS-CoV-2 vaccine, the median anti-spike IgG levels in MM was 38 (IQR, 8-250 AU/mL), significantly lower than those in SMM (650, IQR, 419-1370 AU/mL, p<0.0001, Mann–Whitney U test) and in the control group (995, IQR, 455-1502 AU/mL, p<0.0001, Mann–Whitney U test).

**Figure 1:**
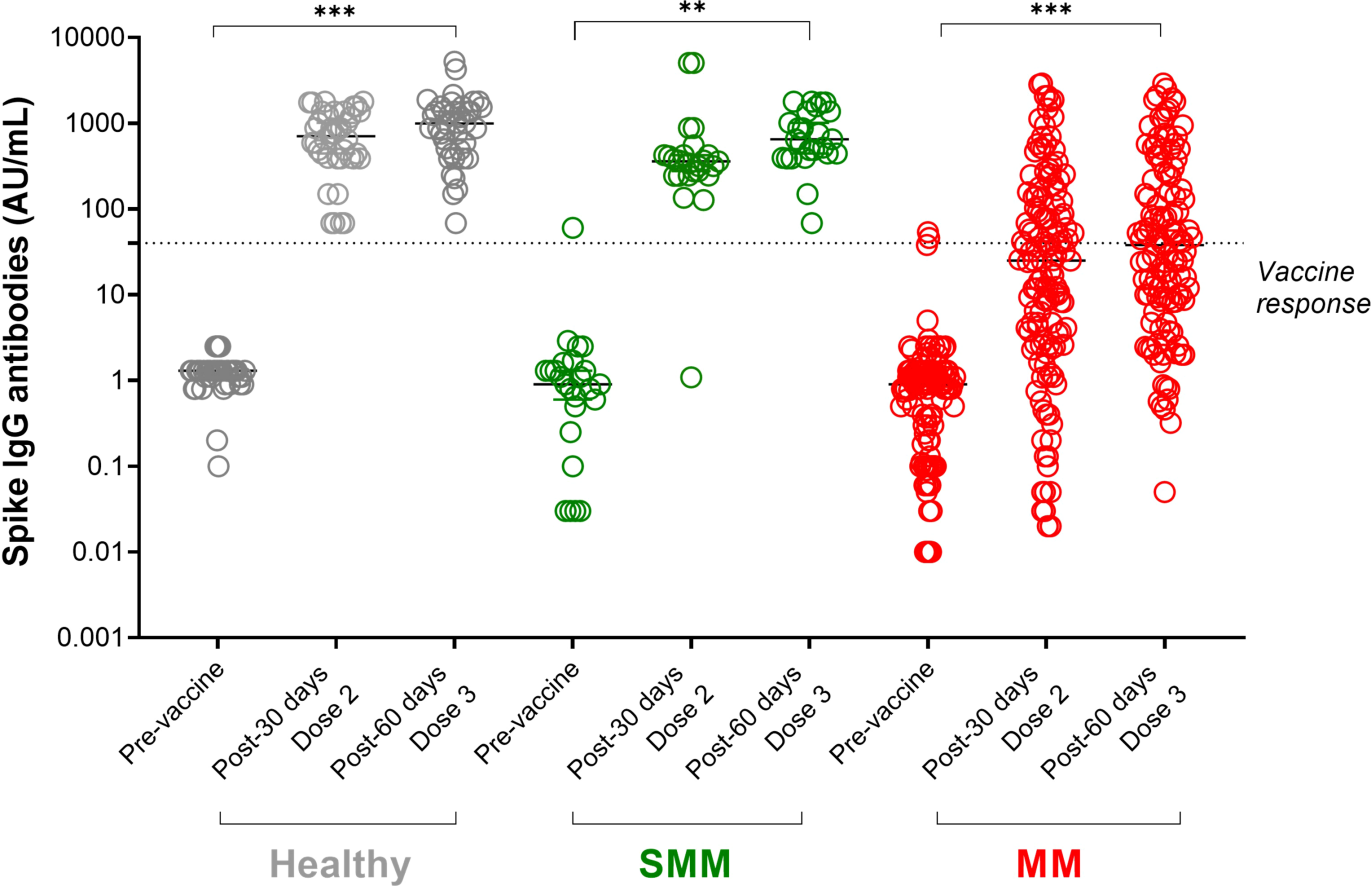
SARS-CoV-2 anti-spike IgG antibody responses in patients with multiple myeloma (MM) before and after a full cycle of COVID-19 RNA vaccination. Spike IgG antibodies in healthy (shown in grey dots), SMM (shown in green dots) and MM (shown in red dots) subjects evaluated at three different timepoints: before, after 30 days from Dose 2 and after 60 days from dose 3 of BNT162b2 (Pfizer-BioNTech) SARS-CoV-2 vaccine. Antibody concentrations measured in artificial units per mL (AU/mL) were depicted on a log-10 scale to better capture the full range of responses. Dotted reference line identifies the threshold at 40 AU/mL corresponding to evidence of vaccine response. Full lines represent the median among the different groups; for each subject category (healthy, SMM and MM) a comparison between pre-vaccine and after each dose is shown. Asterisks denote ANOVA significance among the three timepoints tested: **p<0.001, *** p<0.0001. SMM, smoldering multiple myeloma; MM, multiple myeloma; IgG, immunoglobulins IgG class; AU, arbitrary units.

As shown in **Figure 1**, in each group of subjects, the third dose of SARS-CoV-2 vaccine increased the median of anti-spike IgG levels, with all healthy and SMM subjects reaching a protective titre >40 AU/mL, while 64/133 (48%) MM patients did not reach any protective titre. Among 78 MM patients who did not achieve a vaccine response after a full SARS-CoV-2 vaccine cycle, the third boost dose induced a protective titre only in 12 patients (15%), since 55 patients remained unresponsive and 11 died for MM progression before receiving the boost dose. 121 MM patients further underwent the third injection of a boost dose of anti-SARS-CoV-2 vaccination.

**Figure 2:**
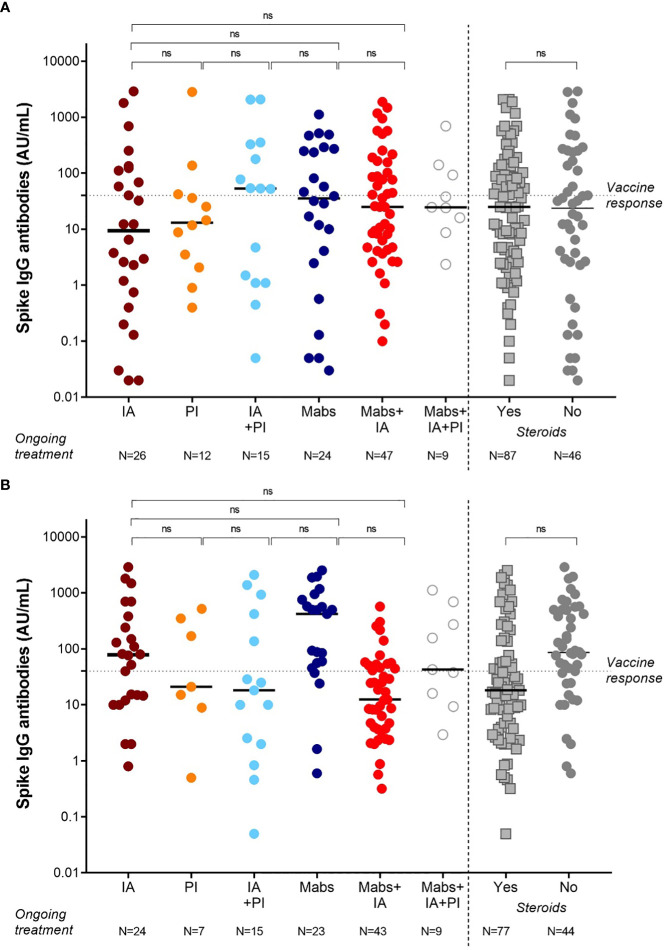
SARS-CoV-2 anti-spike IgG antibody responses in MM patients based on the ongoing treatment after 2 doses **(A)** and 3 doses **(B)** of vaccine BNT162b2 (Pfizer-BioNTech). Antibody concentrations measured in artificial units per mL (AU/mL) were depicted on a log-10 scale to better capture the full range of responses after 2 doses **(A)** and 3 doses **(B)** of vaccine BNT162b2 (Pfizer-BioNTech). Dotted reference line identifies the threshold at 40 AU/mL corresponding to evidence of vaccine response. Full lines represent the median among the different groups; comparisons between different subject categories (IA, PI, Mabs, alone or in combinations) are shown. MM, multiple myeloma; IgG, immunoglobulins IgG class; AU, arbitrary units; IA, immunomodulatory agents (thalidomide, lenalidomide, pomalidomide), PI (proteasome inhibitors, bortezomib, carfilzomib, ixazomib), Mabs (monoclonal antibodies, elotuzumab, daratumumab, belantamab mafodotin); ns, not significant.

**Figure 3:**
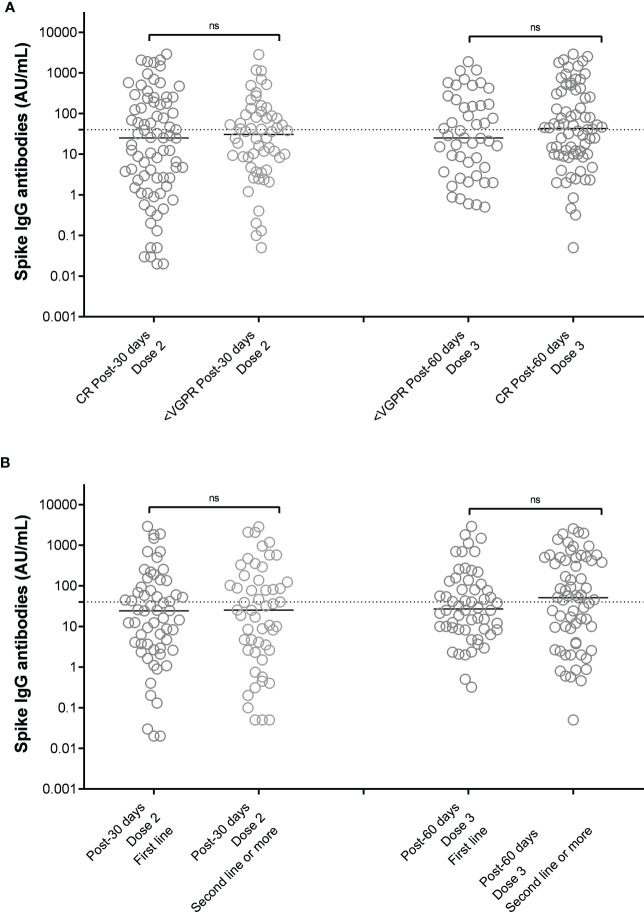
SARS-CoV-2 anti-spike IgG antibody responses after 2 doses of vaccine BNT162b2 (Pfizer-BioNTech) in MM patients distinguished on the basis of response to therapy **(A)** and line of therapy **(B)**. Antibody concentrations measured in artificial units per mL (AU/mL) were depicted on a log-10 scale to better capture the full range of responses after 2 doses and 3 doses of vaccine BNT162b2 (Pfizer-BioNTech), distinguished on the basis of the achieved response to anti-MM therapy at the time of vaccination **(A)** or the treatment line **(B)**. Dotted reference line identifies the threshold at 40 AU/mL corresponding to evidence of vaccine response. Full lines represent the median among the different groups; comparisons between different subject categories are shown. MM, multiple myeloma; IgG, immunoglobulins IgG class; AU, arbitrary units; CR, complete remission based on IMWG criteria, VGPR, very good partial response based on IMWG crieria; ns, not significant.

As summarized in **Figure 2A**, we could not identify a specific drug combination associated with reduced serological response to a complete vaccination cycle. MM patients in continuous treatment with a very good partial response (VGPR) achieved antibody levels (median 25, IQR 2-185 AU/mL) similar to those patients who were not in VGPR (median 31, IQR 5-92 AU/mL, p=0.72, **Figure 3A**).

Based on treatment addressed against MM, the NR-MM were 34/122 (28%) of patients treated with anti-CD38-based immunotherapy, 12/122 (10%) of patients treated with proteasome inhibitors (PI), and 36/122 (30%) of patients treated with immunomodulatory agents. Thus, we failed to identify an association between specific treatment types and reduced vaccine response (**Figure 2B**). Patients on frontline therapy produced the same anti-spike antibody levels (median 24, IQR 3-93, AU/mL) as those achieved in second-line or subsequent treatment (median 25, IQR 2-133 AU/mL; p=0.76; **Figure 3B**).

### 3.3 Clinical predictors of lack of response to SARS-CoV-2 vaccination in MM patients

We defined as non-responders (NR) those subjects who did not achieve anti-spike IgG levels ≥40 arbitrary units per milliliter (AU/ml), identifying 78 (59%) NR patients and 55 (41%) responder patients after the complete vaccination as summarized in **Table 3**. In the NR group there were more patients receiving active treatment (p=0.0003), carrying IgM <50 mg/dL (p=0.003) and high-risk cytogenetics features detected by FISH (p=0.0006).

**Table 3:** Clinical characteristics and treatment overview in 133 MM patients distinguished on serological response to two doses of BNT162b2 (Pfizer-BioNTech) COVID-19 vaccine.

	Non responder N=78	Responder N=55	p-value
Gender, Female, n (%)	32 (58)	23 (42)	0.07
**Disease activity and depth of response, n (%)**			
CR+VGPR	42 (54)	35 (64)	0.25
PR or less	36 (46)	20 (36)	0.25
**Ongoing therapy, n (%)**	41 (98)	37 (79)	***0.0003***
**Previous lines of treatment, n (%)**			
0	37 (47)	23 (42)	0.56
1	29 (37)	23 (42)	0.56
2 or more	12 (15)	9 (16)	0.88
ASCT in the last 12 months	24 (31)	20 (36)	0.55
**Exposure to monoclonal antibodies, n (%)**	46 (59)	33 (60)	0.99
**Ongoing therapy type, n (%)**			
IA	14 (18)	12 (22)	0.57
PI	9 (12)	3 (5)	0.17
Monoclonal antibodies	14 (18)	15 (27)	0.22
IA+PI	9 (12)	6 (11)	0.99
IA+ monoclonal antibodies	27 (34)	15 (27)	0.39
IA+ monoclonal antibodies+ PI	5 (6)	4 (8)	0.99
**Exposure to high-doses dexamethasone**	55 (71)	32 (58)	0.12
**IgM, mg/dL**			
median (IQR)	36 (15-150)	35 (6-134)	0.82
**IgM<50 mg/dL, n (%)**	31 (74)	23 (49)	***0.003***
**ANC, 10^3^ cells/mmc**	3.1	2.9	0.82
median (IQR)	(1.1-12.8)	(1.1-13.3)	
**ALC, 10^3^ cells/mmc**	1.3	1.3	0.82
median (IQR)	(0.1-2.8)	(0.3-3.4)	
**AMC, 10^3^ cells/mmc**	0.5	0.4	0.82
median (IQR)	(0.1-2.8)	(0.1-1.0)	
**FISH-high risk**, N (%)	6 (7.7)	29 (52.7)	***0.0006***

IA, immunomodulatory agents; PI, proteasome inhibitor; CR, complete response; VGPR, very good partial CR; PR, partial response; ASCT, autologous stem cell transplant; ANC, absolute neutrophil count; ALC, absolute lymphocyte count; AMC, absolute monocyte count, IQR, interquartile range.

In bold are reported the variables that achieved a statistically significance.

Following PSM, lack of response to SARS-CoV-2 complete vaccination was associated to age more than 70 years old: adjusted odds ratio (AOR) 2.8, 95% confidence intervals (CI): 1.2-6.5 and high-risk FISH abnormalities (AOR 5.1, 95% CI: 1.9-13.5).

After the complete vaccination cycle plus boost, we identified 64 (48%) NR patients and 57 (52%) responder patients, as summarized in **Table 4**. In the NR group there were more patients receiving continuous treatment (p=0.0003), with monoclonal antibodies, alone (p=0.006) or associated with immunomodulatory agents (p=0.02), high-dose of dexamethasone (p=0.002), carrying IgM <50 mg/dL (p=0.003).

**Table 4:** Clinical characteristics and treatment overview in 121 MM patients distinguished based on serological response to three doses of BNT162b2 (Pfizer-BioNTech) COVID-19 vaccine.

	Non responder N=64	Responder N=57	p-value
Gender, Female, n (%)	27 (42)	21 (37)	0.57
**Disease activity and depth of response, n (%)**			
CR+VGPR	37 (58)	37 (65)	0.43
PR or less	27 (42)	20 (35)	0.43
**Ongoing therapy, n (%)**	59 (92)	53 (93)	0.99
**Previous lines of treatment, n (%)**			
0	33 (52)	23 (40)	0.18
1	23 (36)	24 (42)	0.51
2 or more	8 (12)	10 (18)	0.36
ASCT in the last 12 months	21 (33)	20 (35)	0.92
**Exposure to monoclonal antibodies, n (%)**	40 (63)	34 (60)	0.94
**Ongoing therapy type, n (%)**			
IA	10 (16)	14 (25)	0.22
PI	4 (6)	3 (5)	0.99
Monoclonal antibodies	6 (10)	17 (30)	***0.006***
IA+PI	10 (16)	5 (8)	0.18
IA+ monoclonal antibodies	29 (45)	14 (25)	***0.02***
IA+ monoclonal antibodies+PI	5 (7)	4 (7)	0.99
**Exposure to high-doses dexamethasone**	49 (76)	28 (49)	***0.002***
**IgM, mg/dL**			
median (IQR)	41 (15-150)	95 (6-934)	***0.06***
**IgM<50 mg/dL, n (%)**	47 (73)	45 (79)	0.44
**ANC, 10^3^ cells/mmc**	3.7	3.9	0.72
median (IQR)	(1.1-12.8)	(1.1-13.3)	
**ALC, 10^3^ cells/mmc**	1.2	1.3	0.73
median (IQR)	(0.1-2.8)	(0.3-3.4)	
**AMC, 10^3^ cells/mmc**	0.4	0.5	0.78
median (IQR)	(0.1-2.8)	(0.1-1.0)	
**FISH-high risk, N (%)**	17 (26.5)	10 (17.5)	0.28

IA, immunomodulatory agents; PI, proteasome inhibitor; CR, complete response; VGPR, very good partial CR; PR, partial response; ASCT, autologous stem cell transplant; ANC, absolute neutrophil count; ALC, absolute lymphocyte count; AMC, absolute monocyte count, IQR, interquartile range.

Following PSM, lack of response to SARS-CoV-2 complete vaccination plus boost was associated to age more than 70 years old (AOR 2.8, 95% CI: 1.2-6.6) and use of high-dose of steroids (AOR 3.5, 95% CI: 1.5-7.7).

### 3.4 SARS-CoV-2 breakthrough infections in vaccinated patients and prophylaxed with tixagevimab/cilgavimab

As the pandemic rages on and new SARS-Cov-2 variants emerge, the risk of breakthrough infection has become significant due to the possible immune escape of new variants or the diminished immune response to the three vaccine doses. In our cohort, 20 patients developed breakthrough infections in the first six months after the last inoculation, with the majority not responders to the vaccine (12, 60%), but without any correlation with the IgM value, number of previous lines of therapy, neutropenia or active disease. The disease was characterized by moderate symptoms (fever, flu symptoms, rhinorrhea), with a median duration of positivity to the nasopharyngeal swab of 14 days, with a consequent slight delay in the administration of therapy.

Forty patients with an IgM count lower than 50 mg/dl were treated with intramuscular tixagevimab/cilgavimab 150 mg + 150 mg as prophylaxis without reporting any significant side effects. Nevertheless, during a median follow-up of 90 days, nine patients (22.5%) experienced an infection by SARS-CoV-19. The median days from tixagevimab/cilgavimab administration to infection was 66 days [IQR 62-92]. Of them, one patient experienced the infection for the second time. Five patients (55.6%) had a totally asymptomatic course of the disease, 3 (33.3%) reported only mild symptoms (specifically, flu-like syndrome with cough, hoarseness, fatigue). In contrast, 1 (11.1%) reported fever (no more than 38°C and less than 2 days). Six patients (66.7%) were treated with nirmatrelvir, while 1 with remdesivir. The duration of the infection stood at a median of 10 days (IQR 7-12), leaving no significant sequelae. Six patients delayed the subsequent therapy administration for no more than 8 days (**Table 5**). Following PSM, only the high-risk cytogenetic abnormalities were associated to increased risk to develop a breakthrough infection (AOR 4.2, 95% CI: 1.5-11.8).

**Table 5:** Clinical course of MM’s patients with COVID-19 infection and previously treated with tixagevimab/cilgavimab as prophylaxis therapy.

Patients	Days from tixagevimab/cilgavimab	Prior infection	Clinical course	Days of positivity	Therapies	Sequelae	Days of MM’s treatment delay
1	90	No	Mild symptoms (flu-like syndrome with cough, fatigue)	12	Nirmatrelvir	No	7
2	66	Yes	Asymptomatic	6	Nirmatrelvir	No	7
3	92	No	Asymptomatic	7	Nirmatrelvir	No	0
4	26	No	Asymptomatic	14	Nirmatrelvir	No	7
5	65	No	Asymptomatic	10	None	No	2
6	92	No	Asymptomatic	7	None	No	0
7	158	No	Mild symptoms (flu-like syndrome with fatigue)	13	Nirmatrelvir	No	10
8	52	No	Mild symptoms (flu-like syndrome with cough)	10	Remdesivir	No	7
9	62	No	Fever (1 day), flu-like syndrome	12	Nirmatrelvir	No	7

## 4 Discussion

From the end of January 2020, SARS-CoV-2 spread from China to all countries worldwide and was declared a “SARS-CoV-2 pandemic”. Immunocompromised patients have a higher risk of developing severe events, with improved mortality (9, 10).

The development of vaccines against SARS-CoV-2 represented a central point in the fight against the pandemic, drastically reducing Europe’s expected number of deaths (10). These data are more meaningful in immunocompromised, neoplastic, and frail patients, who enjoy a greater immune defence. Starting from these assumptions, the immunocompromised system in patients with multiple myeloma makes these settings of patients more susceptible to the serious adverse events that could arise with a SARS-CoV-2 infection.

Our findings confirmed that a third mRNA vaccine failed to warrant long-term humoral immune responses against SARS-CoV-2 in the setting of patients treated with high dose of dexamethasone or undergoing continuous MM treatment (with monoclonal antibodies alone or associated with other drugs) or reporting a low value of IgM, as reported by our working group in different hematological diseases (e.g., patients affected by myelofibrosis) (11). In fact, after two months from the third dose, most of the vaccine’s non-responder patients at two doses did not show a stable anti-SARS-CoV-2 titer, highlighting even more the increased risk of mortality in these patients related to the infection, already known from the first evidence during the beginning of the pandemic (12). The Spanish Multiple Myeloma Cooperative group reported 167 patients with MM and SARS-CoV-2 disease outcomes. In-hospital mortality of patients with multiple myeloma was higher (56 patients; 34%) than age-matched and sex-matched patients without cancer (38 patients; 23%) (13).

Our findings, moreover, confirmed the belief that MM patients, although responding to the SARS-CoV-2 vaccination, present a lower response compared to those affected by smoldering myeloma and the healthy control group, due to immune system impairment for intrinsic or therapy-related immunosuppression, age, stage of pathology, and other comorbidities (13). However, our study also confirms that SARS-CoV-2 vaccination proved to be highly effective at preventing severe illness in all age groups in the first studies, also holding up that protection after six months (14).

At the end of 2021, there was emerging evidence that giving a third boost dose of the SARS-CoV-2 vaccine could effectively prevent severe illness caused by the SARS-CoV-2 B.1.617.2 (Delta) variant in the population (15, 16). A further coronavirus wave affected our patients at the beginning of January 2022 with the currently dominant SARS-CoV-2 B.1.1.529 (Omicron) variant, when the efficacy of the third dose was largely unknown in patients with hematologic malignancies (17).

In an extensive series of 476 MM patients, the serological response after one week from the third dose injection in patients who did not develop SARS-CoV-2 resulted in significant increases of anti-S IgG across all treatment groups, including in patients receiving an anti-CD38 or anti-BCMA-targeted therapy (18). Our cohort did not confirm the same results underlying the role of IgM value, high-dose steroid therapy, and continuous treatment for developing an adequate antibodies response in this setting of immunocompromised patients.

The inability of the third dose of vaccine to induce a good antibody response suggests that a fourth dose might be similarly ineffective, unlike in normal subjects. For this reason, there has recently been an indication to treat immunocompromized subjects with the prophylactic administration of tixagevimab/cilgavimab. This monoclonal antibody can avoid viral entry by inhibiting the viral spike protein attachment to the surface of the cells, administered through two concomitantly intramuscular injections (19). The efficacy of the treatment was evaluated with the PROVENT study, featuring over 5000 subjects, showing that tixagevimab/cilgavimab reduced the risk of SARS-CoV-2 infection by 77%, with the duration of protection from the virus estimated to be at least six months (20). We chose to use this prophylaxis (a single 150/150 mg dose) in 40 patients we considered to be at higher risk because of IgM reduction. Twenty-two% of patients still contracted SARS-CoV-2 infection after a median time of 66 days, but the course of this infection was very mild. This incidence seems somewhat higher than in other studies reported in the literature on hematologic patients. In fact, a study of 52 patients with a median follow-up of only 79 days reported a 4% incidence of breakthrough infections after prophylaxis with tixagevimab/cilgavimab (21) and another more recent study of 251 patients, treated with a double dose of monoclonal antibody (300/300 mg), documented an incidence of breakthrough infections of 11% (22). However, these studies included several hematologic diseases, and there were only 8 and 32 myeloma patients in the two studies. Our study focused instead on myeloma patients and, to our knowledge, is the first to evaluate the effectiveness of this type of prophylaxis on a cohort consisting exclusively of myeloma patients. Regardless of the incidence rates of breakthrough infections, our study confirms that prophylaxis with tixagevimab/cilgavimab has a significant protective effect in that none of our patients had a severe SARS-CoV-2 infection although all were significantly immunocompromised.

Therefore, tixagevimab/cilgavimab may be a handy tool in patients with peculiar conditions who did not respond to SARS-CoV-2 vaccination (evaluating the anti-SARS-CoV-2 antibody serum titer) to prevent severe and symptomatic infection, with a reduced duration of the infection, no sequelae, and a limited delay of furthering treatments. The role of prophylaxis should be better addressed with studies enrolling a large series of patients, allowing these patients to receive passive immunization with tixagevimab/cilgavimab instead of the fourth dose of vaccine to warrant long-term protection.

## 5 Conclusion

MM patients are highly vulnerable, and emerging data indicate that a third mRNA vaccine failed to warrant long-term humoral immune responses against SARS-CoV-2, showing an unstable anti-SARS-CoV-2 titer after two months from the third dose.

This study highlights the need for moving the attention to monitoring immune responses, confirming that in the setting of immunosuppressed patients, the use of prophylaxis with tixagevimab/cilgavimab can guarantee better protection from SARS-CoV-2 by reducing the risk of serious disease.

## Data availability statement

The raw data supporting the conclusions of this article will be made available by the authors, without undue reservation.

## Ethics statement

The studies involving human participants were reviewed and approved by Ethical Committee CT1/A.O.U. Policlinico – Catania. The patients/participants provided their written informed consent to participate in this study.

## Author contributions

AR, AD and CC performed the research. DL, EL, DC, DT and CG collected data and processed samples. AR, AB, AD, VD and GP contributed to data interpretation and data analysis. AR, DL, and AD wrote the paper. AR, CC and FR revised the final manuscript. All authors contributed to the article and approved the submitted version.
